# A55 INTERLEUKIN-10 ELICITS CYTOPROTECTION VIA MITOCHONDRIAL SIGNAL TRANSDUCER AND ACTIVATORS OF TRANSCRIPTION 3 (MTSTAT3) TO PREVENT BACTERIAL PATHOBIONT EVOKED MITOCHONDRIAL FRAGMENTATION IN GUT EPITHELIA

**DOI:** 10.1093/jcag/gwab049.054

**Published:** 2022-02-21

**Authors:** S NavaneethaKrishnan, A Wang, D M Mckay

**Affiliations:** 1 Physiology and Pharmacology, University of Calgary, Calgary, AB, Canada; 2 Physiology & Pharmacology, Uni. Calgary, Calgary, AB, Canada

## Abstract

**Background:**

Aberrant mitochondrial function is increasingly reported in inflammatory bowel disease (IBD). We recently reported that the IBD-associated pathobiont, adherent-invasive *E. coli* (AIEC) (strain LF82)-evoked epithelial mitochondrial fragmentation and mitochondrial depolarization. Remarkably, the transcription factor, signal transducer and activator of transcription (STAT)-3, can migrate to mitochondria (mtSTAT3) to regulate functions such as reactive oxygen species and ATP production. This non-canonical activity is dependent on STAT3 phosphorylation at serine727. IL-10 is an important regulator of enteric homeostasis that signals via STAT-3, however whether IL-10 affects mitochondrial dynamics is unknown.

**Aims:**

To determine (1) if IL-10 preserves mitochondrial function in *E. coli*-LF82-infected epithelia through mtSTAT3, and if so, (2) to identify the mechanism by which mtSTAT3 restores mitochondrial functions.

**Methods:**

The human colon-derived T84 epithelial cells or human organoids were exposed to *E. coli* LF82 (10^8^ cfu/ml, 4h) ± co-treatment or an 18h pre-treatment with IL-10 (10 ng/ml). The effect of IL-10 on bacterial growth and its invasion of T84 cells were assessed by growth curve analysis and bacterial internalisation assays. We assessed mitochondrial network morphology using mito-tracker red and confocal microscopy, mitochondrial membrane potential with the fluorescent-dye TMRE and flow cytometry and fluorescence microscopy, and oxygen consumption rate (OCR) and ATP levels. STAT3 phosphorylation was analysed in whole cell protein extracts by western blot. Pharmacological inhibitors of JAK and Erk1/2 were used to examine the pathway of mtSTAT3 activation.

**Results:**

IL-10 affected neither *E. coli*-LF82 growth nor invasion of T84 epithelia, but substantially reduced the AIEC-induced mitochondrial fragmentation in T84 cells and organoids. IL-10 preservation of the mitochondrial network was accompanied by increased mitochondrial membrane potential, OCR, and ATP levels in *E. coli*-LF82 infected T84 cells. IL-10 + *E. coli*-LF82-treated epithelia displayed increased phospho-S^727^ STAT3 that was reduced by inhibition of Erk1/2 but not JAK activity.

**Conclusions:**

Mitochondria are a potential drug target in IBD, and the *E. coli*-LF82 pathobiont can be added to a growing list of microbial pathogens that evoke epithelial mitochondrial dysfunction. We have uncovered that IL-10 can stabilize mitochondrial function in *E. coli*-LF82 infected cells, possibly through mtSTAT-3. Our results underscore the role of IL-10/STAT3 signaling in preserving mitochondrial functions that could result in mitochondria-targeted therapeutics in IBD and other bacteria-driven enteropathies.

**Funding Agencies:**

CIHRAIHS

